# Partially Coherent, Radially Polarized Beam with Annular Apodization

**DOI:** 10.1155/2014/160945

**Published:** 2014-01-19

**Authors:** C. Mariyal, P. Suresh, K. B. Rajesh, T. V. S. Pillai

**Affiliations:** ^1^Department of ECE, National College of Engineering, Tirunelveli, Tamilnadu 627007, India; ^2^Department of Physics, Chikkanna Government Arts College, Tirupur, Tamilnadu 641602, India; ^3^Department of Physics, University College of Engineering, Nagercoil, Tamilnadu 629002, India

## Abstract

Based on the vectorial Debye theory, the tight focusing properties of partially coherent, radially polarized vortex beams are investigated in detail. In this paper, we propose to use an amplitude modulated filter in combination with a high NA lens to generate long focal depth in the focal region. Numerical results show that the generation of long focal depth of FWHM (22.08*λ*) is achieved, which finds useful application in microscopy techniques such as particle acceleration, laser processing, optical micromanipulation, and beam shaping.

## 1. Introduction

In recent years, the partially coherent light under tight focusing finds huge applications such as optical communication, optical sensors, optical data storage, optical manipulation, microscopy, material processing, microparticle trapping manipulation, and optical measuring instruments [1–8]. Recently, several groups have explored the properties of optical vortex formed in partially coherent light both theoretically and experimentally. In 1998, Gori et al. constructed partially coherent beams carefully to carry optical vortex modes theoretically [9–11] and experimentally by Bogatyryova et al. [12]. Richards and Wolf investigated the focusing properties of incident linearly polarized beam by a high NA lens, based on vectorial diffraction theory [13]. Nowadays, the cylindrical vector beam has attracted very much attention due to its unique properties under tight focusing [14–18]. Recently, Youngworth and Brown reported that the tightly focused radially polarized beams produce a tighter spot with a strong longitudinal component and that azimuthally polarized beams produce a hollow light spot [14]. The partially coherent light has universality in its characteristics, so it is important to investigate the radially polarized partially coherent beams [19, 20]. However, no detailed studies were available on the tight focusing effect of partially coherent beams on the high NA focusing objective lens. Recently, Guo et al. [21] studied the tight focusing properties of partially coherent radially polarized vortex beams. Most of these near field applications demand subwavelength beam with a large depth of focus (DOF) and high resolution. A lot of optical methods to improve the resolution limit and the depth of focus were extensively investigated using amplitude apertures [22, 23], phase apertures [23], or their combination [24, 25] in the last few years. In this paper, we investigate the tight focusing properties of amplitude modulated radially polarized partially coherent vortex beam that is tightly focused by a high NA lens based on the vector diffraction theory. The numerical result shows that one can generate an optical needle in the focal region of an incident beam with amplitude modulated filter which is very much useful for optical micromanipulation applications.

## 2. Theory

We assume that the field amplitude in the source plane is a Gaussian model with an optical vortex that can be expressed as [26]

(1) $$\begin{array}{c} W\left(r_{1},r_{2},0\right)=A\left(r_{1},r_{2}\right)\exp\left[in\left(\varphi_{2}-\varphi_{1}\right)\right], \end{array}$$

where

(2) $$\begin{array}{c} A\left(r_{1},r_{2}\right)=\exp\left[-\frac{\left(r_{1}^{2}+r_{2}^{2}\right)}{\omega_{0}^{2}}\right]\exp\left[-\frac{\left(r_{1}^{2}-r_{2}^{2}\right)}{L_{c}^{2}}\right], \end{array}$$

where *L*_*c*_ is the source coherence length.

Under condition *r* = *f*sin*θ*, where *f* is the focal length of the objective, the cross-spectral density of such a partially coherent vortex beam of the pupil can be expressed as

(3) A(θ1,θ2)=exp[−f2(sin2θ1+sin2θ2)ω02]×exp[−f2(sin2θ1−sin2θ2)Lc2].

When a completely coherent radially polarized vortex beam is focused through a high NA objective lens, the total electric field in the focal region can be expressed as [25–28]

(4) $$\begin{array}{c} E\left(r,\psi ,z\right)=-i^{n+1}E_{0}\left[\begin{array}{c} E_{x}\left(r,\psi ,z\right)\\ E_{y}\left(r,\psi ,z\right)\\ E_{z}\left(r,\psi ,z\right) \end{array}\right],\\ E\left(r,\psi ,z\right)=-i^{n+1}E_{0}\left[\begin{array}{c} \left(i\left(I_{n+1}e^{i\psi }-I_{n-1}e^{-i\psi }\right)\right)\\ \left(I_{n+1}e^{i\psi }+I_{n-1}e^{-i\psi }\right)\\ \left(2I_{n}\right) \end{array}\right]\text{ex}{\text{p}}^{in\psi }, \end{array}$$

where *r*, *ψ*, and *z* are the cylindrical coordinates of an observation point in the focal region, *E*_0_ is a constant, and *n* is the topological charge, where

(5a) In(r,z)=∫0αP(θ)cosθsin2θJn(krsinθ)×exp(ikzcosθ)dθ,

(5b) In±1(r,z)=∫0αP(θ)cosθsinθcosθJn±1(krsinθ)×exp(ikzcosθ)dθ,

where *P*(*θ*) is the pupil apodization function and *J*_*n*_ is the *n*th order Bessel function of the first kind. Assuming that the field wave is monochromatic, the cross-spectral density matrix of partially coherent beams is given by [30]

(6) Wij(r1,r2)=〈Ei∗(γ1,ψ1z1)Ej(γ2,ψ2z2)〉,where  (i,j=x,y,z),

where *E*_*i*_(*γ*_1_, *ψ*_1_, *z*_1_) and *E*_*j*_(*γ*_2_, *ψ*_2_, *z*_2_) denote the Cartesian components of the electric field, the asterisk stands for the complex conjugate, and the angle brackets represent an ensemble average.

The explicit expressions of the diagonal elements of *W*_*ij*_ can be derived from (1) as follows:

(7a) Wxx(r1,rz,z)=E02[In+1∗(r1,z)e−iψ1−In−1∗(r1,z)eiψ1]×[In+1(r2,z)e−iψ2−In−1(r2,z)eiψ2]×exp[in(ψ2−ψ1)],

(7b) Wyy(r1,rz,z)=E02[In+1∗(r1,z)e−iψ1+In−1∗(r1,z)eiψ1]×[In+1(r2,z)eiψ2+In−1(r2,z)e−iψ2]×exp[in(ψ2−ψ1)],

(7c) $$\begin{array}{c} W_{zz}\left(r_{1},r_{z},z\right)=4E_{0}^{2}I_{n}^{*}\left(r_{1},z\right)I_{n}\left(r_{2},z\right)\exp\left[in\left(\psi_{2}-\psi_{1}\right)\right], \end{array}$$

(8) Ip∗(r1,z)Iq(r2,z)  =∬0αA(θ1,θ2)cosθ1cosθ2sinθ1sinθ2g(θ1)g(θ2)×Jp(kr1sinθ1)Jq(kr2sinθ2)×exp[ikz(cosθ2−cosθ1)]dθ1dθ2,

where

(9) g(θi)={sinθ,p,q=n,cosθ,p,q=n±1.

The intensity distribution *I*(*r*, *ψ*, *z*) of the focal field in the focal region is given by [30, 31]

(10) I(r,ψ,z)=TrW(r,ψ,z)=Wxx(r,ψ,z)+Wyy(r,ψ,z)+Wzz(r,ψ,z),

where *Tr* denotes the trace of the electric cross-spectral density matrix *W*(*r*_1_, *r*_2_).

## 3. Result

In this paper, we describe a numerical study in the focal region of incident, partially coherent, radially polarized vortex beam based on vector diffraction theory that is tightly focused by a combination of proposed amplitude modulated filter and a high NA lens [13]. Without loss of validity and generality, it was supposed that NA = 0.95, *λ* = 1, and *E*_0_ = 1 for simplicity. To illustrate the axial intensity distribution and the associated focal depth, numerical calculations were performed. The numerical calculation is performed for the topological charge *n* = 1; it can be seen that a rotationally symmetric and tiny dark core with nonzero intensity is surrounded by a high-intensity ring in the focal plane.

**Figure 1:**
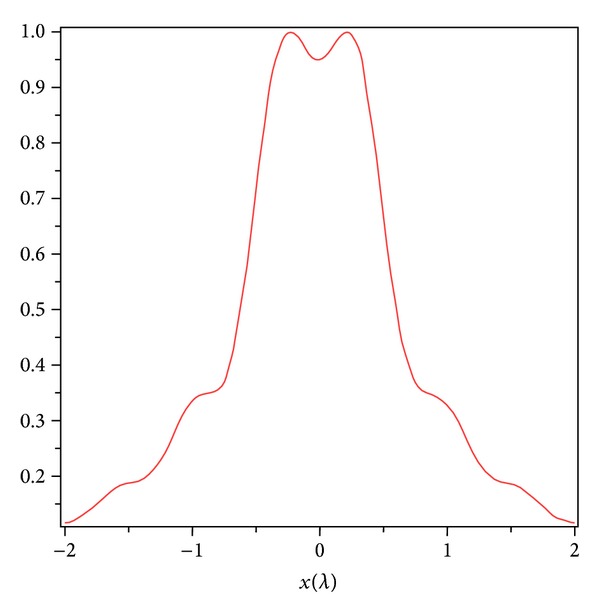
Two-dimensional intensity distribution of a partially coherent, radially polarized vortex beam for *L*_*c*_ = 0.1 cm, *ω*_0_ = 1 cm, *f* = 1 cm, *n* = 1, and *α* = 70°.

Firstly, based on (10), the normalized two-dimensional intensity distributions in focal region of the focused beam are investigated numerically and are illustrated in Figure 1 for *L*_*c*_ = 0.1 cm, *α* = 70°, and it agreed with the result shown in Figure  2(a) of [21]. It should be noted that the distance unit in all figures in this paper is *λ*, where *k* is the wave number (*k* = 2*π*/*λ*). Here, *α* is the convergence semiangle of the lens such that *α* = arcsin(NA/*n*), NA is the numerical aperture, and *n* is the index of refraction between the lens and the sample.

**Figure 2:**
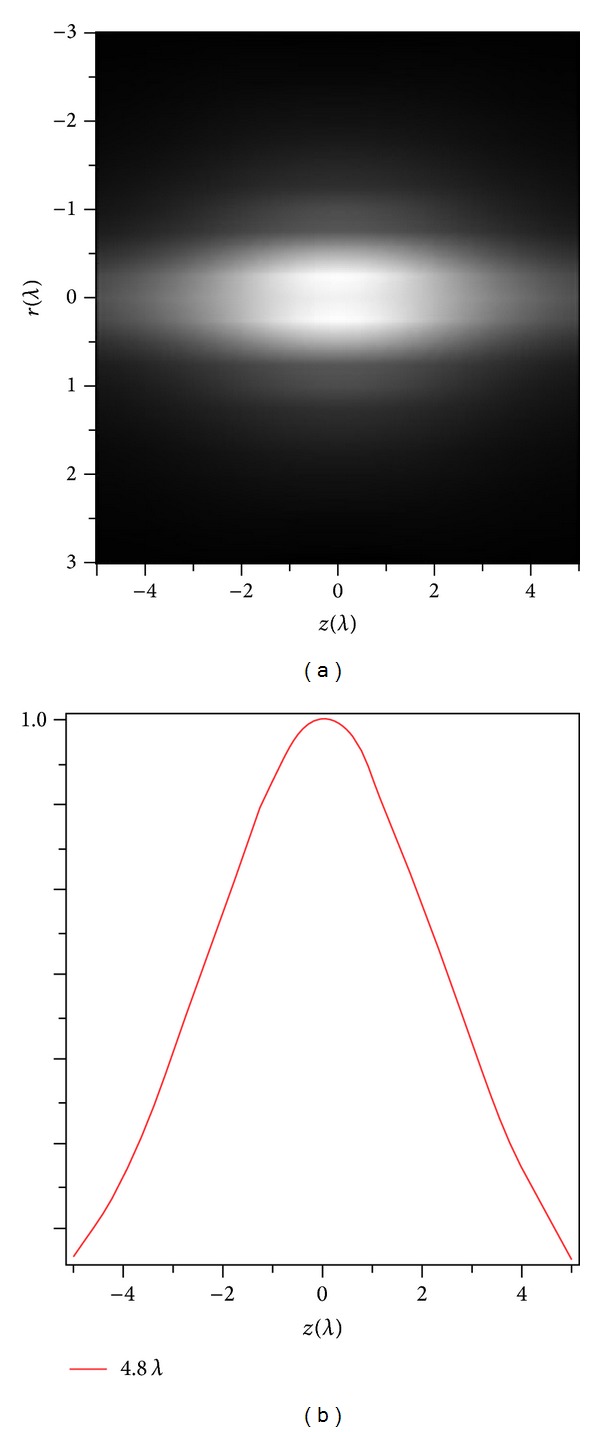
Intensity distribution of the partially coherent radially polarized vortex beam of high NA lens for NA = 0.95, other parameters are the same as in Figure 1.

Figure 2 shows the normalized total electric field intensity distribution in the focal region of high NA objective lens under the illumination of partially coherent, radially polarized vortex beams for NA = 0.95. The other parameters are the same as in Figure 1.  Figure 2(a) shows that three-dimensional total electric field intensity distribution in the focal region of incident beam generates a focal depth of FWHM (4.8 *λ*) and that its corresponding two-dimensional intensity distribution at *r* = 0 is shown in Figure 2(b). However, the focal depth of the incident beam is smaller in the focal region. To expand the depth of focus in the focal region of incident, partially coherent, radially polarized vortex beams, we propose to use diffractive optical element (DOE).

In order to study the effect of DOE, we replaced *A*(*θ*_1_, *θ*_2_) by *A*_0_(*θ*_1_, *θ*_2_)*T*_0_(*θ*_1_, *θ*_2_), it is necessary to increase the concentric rings of the DOE to increase the depth of focus in the focal region. The intensity distribution of the modified DOE with six concentric rings of the input beam can be calculated by rewriting the apodization function of (2) which is rewritten as

(11) $$\begin{array}{c} A\left(\theta_{1},\theta_{2}\right)=A_{0}\left(\theta_{1},\theta_{2}\right)T_{0}\left(\theta_{1},\theta_{2}\right), \end{array}$$

where

(12) $$\begin{array}{c} T_{0}\left(\theta_{1},\theta_{2}\right)=\{\begin{array}{cc} 0, & 0\leq \theta \leq \delta_{1},\;\;\delta_{2}\leq \theta \leq \delta_{3},\;\;\delta_{4}\leq \theta \leq \delta_{5},\\ 1, & \delta_{1}\leq \theta \leq \delta_{2},\;\;\delta_{3}\leq \theta \leq \delta_{4},\;\;\delta_{5}\leq \theta \leq \alpha , \end{array} \end{array}$$

where

(13) $$\begin{array}{c} \delta_{r}=R_{r}\cdot \alpha ,\;\text{where}\;\;r=1,2,\ldots ,5. \end{array}$$

We choose one structure with random values for *δ*_1_ to *δ*_5_ from all possibilities and simulate their focusing properties by vector diffraction theory. If the structure generates a subwavelength long focal depth and satisfies the limiting conditions of side lobe intensity of less than 15%, it is chosen as the initial structure during the optimization procedures. In the following steps, we continue to vary *R* of one chosen zone to generate a long focal depth on an optical axial electric field until the value of the focal depth is not getting smaller or the focusing properties are not satisfying the limiting condition. The value of the newly chosen zone thickness is used in the next step. Then, we randomly choose the other zone and repeat these procedures to improve the uniformity of the on-axial intensity profile without affecting the limiting condition. We repeat these procedures and, as an example, the sets of optimized “*R*” values to generate long focal depth in the focal segment of the high NA objective lens are *R*_1_ = 0.09, *R*_2_ = 0.32, *R*_3_ = 0.71, *R*_4_ = 0.82, and *R*_5_ = 0.95.

**Figure 3:**
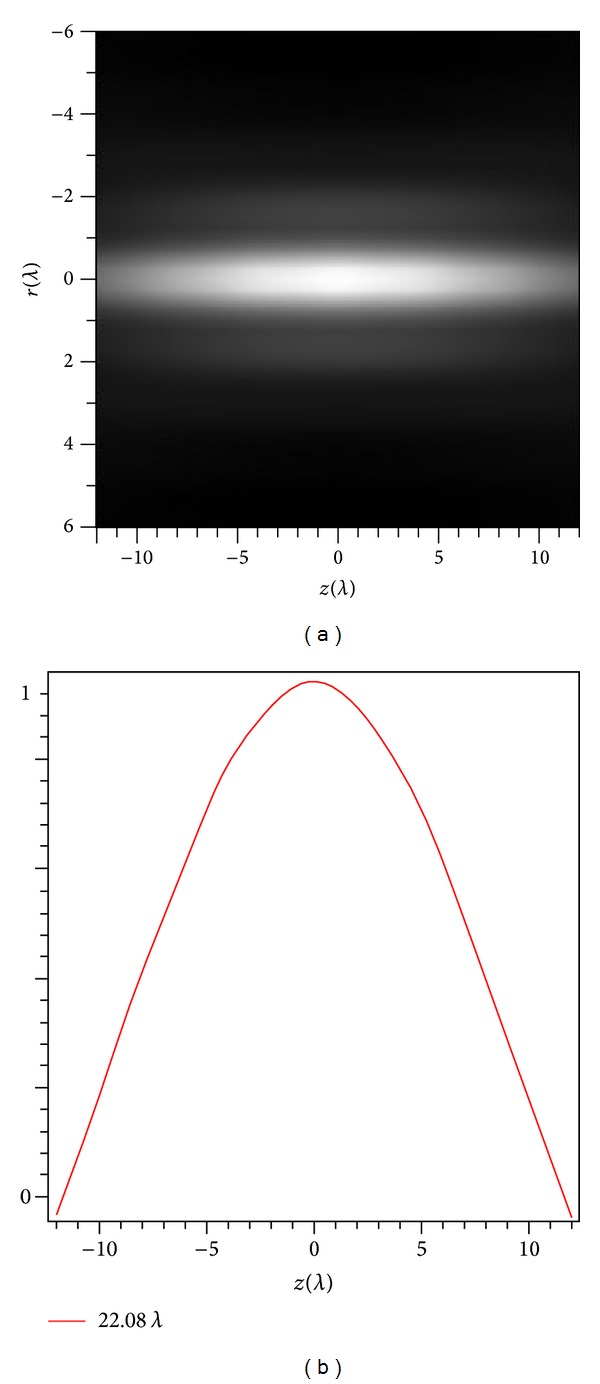
Intensity distribution of the partially coherent, radially polarized vortex beam of high NA lens with amplitude modulated filter for NA = 0.95, other parameters are the same as in Figure 1.

With appropriate combinations of these adjustments (*R*_*r*_), an optical needle (“long focal depth”) can be generated in the focal region of high NA lens as it is shown in Figure 3.

Figure 3 shows the normalized total electric field intensity distribution in the focal region of high NA objective lens under the illumination of partially coherent, radially polarized vortex beams for NA = 0.95. The other parameters are the same as in Figure 1.  Figure 3(a) shows that three-dimensional total electric field intensity distribution in the focal region of incident beam generates a focal depth of FWHM (22.08*λ*) and that its corresponding two-dimensional intensity distribution at *r* = 0 is shown in Figure 3(b). We observed that the generated focal segment in the focal region in combination with DOE of incident beam is 4.6 times greater which is suitable and has high resolution for the above applications.

## 4. Conclusion

We have studied the tight focusing effect of incident, partially coherent, radially polarized beams in the focal field of high NA lens with DOE numerically. The Numerical results show that the generation of long focal depth of FWHM (22.08*λ*) is achieved in the focal region high NA lens in combination with DOE, which finds useful application in microscopy techniques such as particle acceleration, laser processing, optical micromanipulation, and beam shaping.
